# Oxidative Modifications of RNA and Its Potential Roles in Biosystem

**DOI:** 10.3389/fmolb.2021.685331

**Published:** 2021-05-12

**Authors:** Mikiei Tanaka, P. Boon Chock

**Affiliations:** Biochemistry and Biophysics Center, National Heart Lung and Blood Institute, National Institutes of Health, Bethesda, MD, United States

**Keywords:** translation error, oxidative stress, 8-oxoguanine, abasic site, apoptosis, signal transduction pathway, cross-link, inflammation

## Abstract

Elevated level of oxidized RNA was detected in vulnerable neurons in Alzheimer patients. Subsequently, several diseases and pathological conditions were reported to be associated with RNA oxidation. In addition to several oxidized derivatives, cross-linking and unique strand breaks are generated by RNA oxidation. With a premise that dysfunctional RNA mediated by oxidation is the pathogenetic molecular mechanism, intensive investigations have revealed the mechanism for translation errors, including premature termination, which gives rise to aberrant polypeptides. To this end, we and others revealed that mRNA oxidation could compromise its translational activity and fidelity. Under certain conditions, oxidized RNA can also induce several signaling pathways, to mediate inflammatory response and induce apoptosis. In this review, we focus on the oxidative modification of RNA and its resulting effect on protein synthesis as well as cell signaling. In addition, we will also discuss the potential roles of enzymatic oxidative modification of RNA in mediating cellular effects.

## Introduction

Reactive oxygen species (ROS) and free radicals play major roles in normal biological functions. However, they can also react rapidly with biomacromolecules, such as nucleic acids, proteins, or lipids, and alter their biological functions. Therefore, they have been implicated in a wide range of age-related neurodegenerative diseases and aging (*See* (Stadtman and Berlett, 1997; Beckman and Ames, 1998; Thannickal and Fanburg, 2000) for Rev.). With respect to nucleic acid oxidation, DNA is known to be protected by its binding proteins, and by a number of repair systems to minimize its damaging effects (Evans et al., 2004). However, RNAs are reportedly more susceptible to oxidation than DNA (Hofer et al., 2005). As a result, age-related diseases would be more likely to be associated with RNA oxidation. Consistent with this notion, an elevated level of oxidized RNA was found in Alzheimer’s disease (AD) (Nunomura et al., 1999), and Parkinson’s disease (PD) (Zhang et al., 1999). Consequently, research on RNA oxidation has been directed toward understanding the potential mechanisms by which oxidized RNA mediates age-related diseases. To date, elevated oxidation of RNA, monitored by the formation of 8-oxo-7,8-dihydroguanosine (8-oxo-G), has been reported in a variety of diseases such as diabetes (Cejvanovic et al., 2018), schizophrenia (Jorgensen et al., 2013), and depression (Jorgensen et al., 2013). The fact that 8-oxo-G rather than 8-oxo-7,8-dihydroxydeoxyguanosine (8-oxo-dG) was found elevated in the urine or plasma of disease patients, suggests that the disease is associated with the elevation of RNA oxidation. Therefore, monitoring the extent of RNA oxidation *via* its oxidative stress biomarker provides a useful method for diagnosing age related diseases (*see* review (Poulsen et al., 2012; Jacob et al., 2013; Larsen et al., 2019)). However, the molecular analysis of RNA oxidative modifications and cellular responses have not been fully investigated. Based on DNA oxidation studies (Lindahl, 1993), 8-oxo-G and abasic site, which is the ribose moiety after depurination/depyrimidination, are most likely to be the predominant oxidative derivative of ribonucleosides. In addition, RNA oxidation could lead to the formation of cross-linking with proteins and a strand scission. From the pathogenetic point of view, we and others have reported the effects on translational activity mediated by the oxidation of tRNA (Preiss et al., 1959; Sochacka et al., 2015), mRNA (Shan et al., 2007; Tanaka et al., 2007; Calabretta et al., 2015; Thomas et al., 2019), and rRNA (Honda et al., 2005; Willi et al., 2018). The decrease in translational activity is due to the accumulation of aberrant polypeptides caused by premature termination and amino acid misincorporation. Together, these could constitute a pathogenic mechanism, since accumulation of abnormal proteins is known to disrupt protein homeostasis, a phenomenon observed in many pathological features in diseases (Hipp et al., 2014). On the other hand, recent extensive research on the RNA quality control systems revealed that dysfunctional mRNA including oxidized mRNA, in which ribosomes are stalled or collided would be eliminated together with nascent polypeptides by a set of proteins including endoribonuclease and ubiquitin ligase (*see* review (Ikeuchi et al., 2018; Collart and Weiss, 2020)). However, the regulatory mechanisms mediated by oxidized RNA per se are not fully investigated.

**FIGURE 1:**
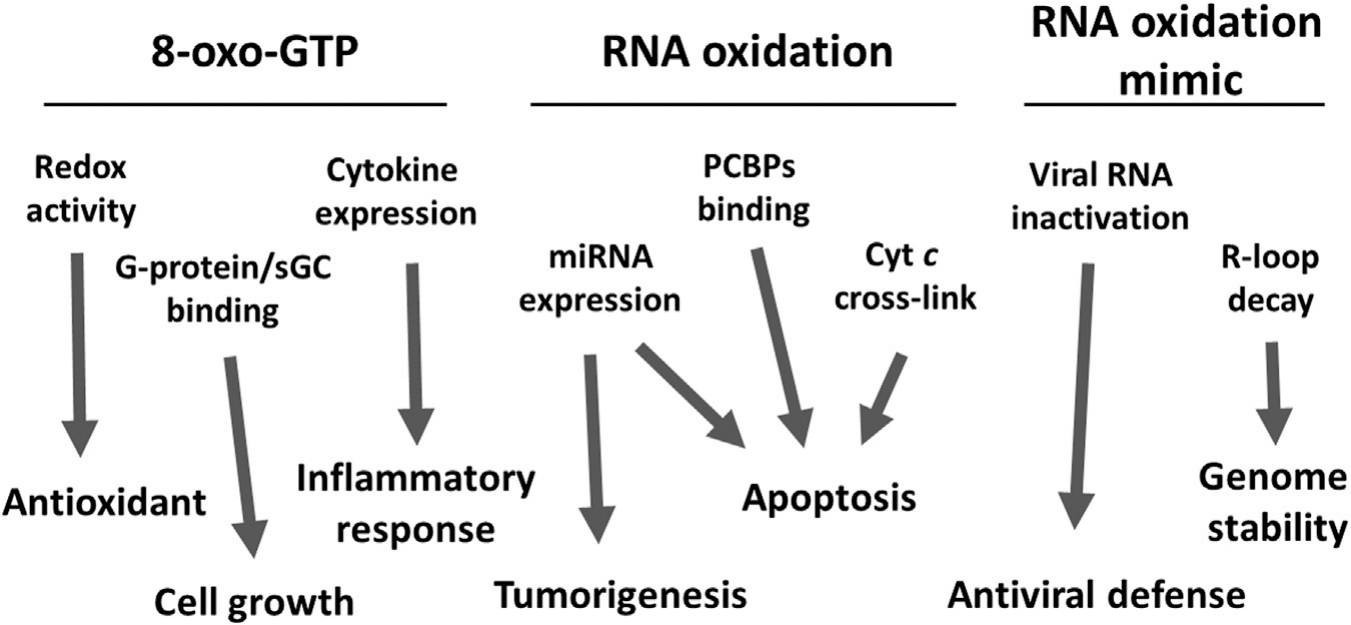
A schematic diagram of the potential roles of oxidized RNA. Three types of oxidized RNA, free 8-oxo-GTP, oxidized RNA, and the equivalently modified RNA modulate several cellular functions and signaling pathways. The possible effects of those modulations, but not limited to are also represented. The detailed mechanisms are described in the text.

ROS are known to be involved in mediating cell signaling (Rhee, 2006; Bochkov et al., 2010; Alenko et al., 2017; Lanz et al., 2019; Sengupta et al., 2020). In line with this notion, it has been reported that oxidatively modified RNAs could interact with various biological molecules known to mediate cell signaling. In addition to the negative impacts caused by compromising the RNA biological functions, RNA oxidation could also modulate certain cell signaling pathways. Evidence has emerged showing the products of RNA oxidation, *e.g.* 8-oxo-G as well as 8-oxo-dG could exhibit an antioxidant function (Lee et al., 2013). In addition, 8-oxo-GTP has been reported to modulate G-protein GTPase activity (Yoon et al., 2005) and soluble guanylyl cyclase (Bolin and Cardozo-Pelaez, 2009). Furthermore, oxidation of mitochondrial RNA could modulate the inflammatory response through cytokine induction (Saxena et al., 2017). RNA containing the 8-oxo-G could modulate an apoptotic signaling pathway mediated by the oxidized RNA’s binding proteins (Ishii et al., 2020). In addition, an oxidatively modified microRNA such as miR-184 has been shown to mediate cellular apoptosis via its interaction with the 3-UTR region of Bcl-xL and Bcl-w (Wang et al., 2015), and processing of miRNAs such as miR-221 has been shown to be regulated *via* oxidative modification and the apurinic/apyrimidinic endoribonuclease1 (APE1) (Antoniali et al., 2017). We have shown that cytochrome *c* (cyt *c*) could form a cross-linked complex with oxidized RNA, and facilitate its dissociation from mitochondria to the cytosol, a process required to initiate the mitochondria-mediated apoptosis. Our findings imply that oxidative modification of RNA facilitates cellular apoptosis *via* a protective signal in response to oxidative stress (Tanaka et al., 2012). In addition, in this review, we will also describe other possible roles of enzyme-catalyzed, oxidation-like modifications of RNA. In this case, the formation of the RNA abasic site is catalyzed by an N-glycosylase which in turn is processed by APE1 in the R-loop region to modulate DNA replication or transcription possibly (Liu et al., 2020). Pokeweed antiviral protein initiates an antiviral defense by abasic formation of viral RNAs (Di and Tumer, 2015). As illustrated in Figure 1, oxidized RNA could exert extensive biological functions, a subject that needs further investigation.

## RNA Oxidative Modifications

Among endogenous ROS, superoxide anions and H_2_O_2_ are ubiquitously generated from the electron transport chain (Boveris and Chance, 1973; Boveris and Cadenas, 1975). A superoxide anion is converted to H_2_O_2_ in a reaction catalyzed by superoxide dismutase. While the reactivity of both the superoxide anion and H_2_O_2_ are relatively mild, in the presence of metal ion such as Fe(II), H_2_O_2_ is converted to hydroxyl radical, which is a highly reactive ROS (Goldstein et al., 1993; Wardman and Candeias, 1996). This metal ion catalyzed hydroxyl radicals generation is known as the Fenton reaction, a reaction thought to be the most pathophysiologically relevant in mediating RNA oxidation (Nunomura et al., 1999; Honda et al., 2005). Since RNA contains multiple high affinity binding sites for iron, some of these irons are likely to bind to the Mg(II) binding sites (Berens et al., 1998; Athavale et al., 2012; Zinskie et al., 2018). Hereafter, we primarily consider the RNA oxidation proceeds *via* iron mediated Fenton reaction.

### 8-oxo-G

**FIGURE 2:**
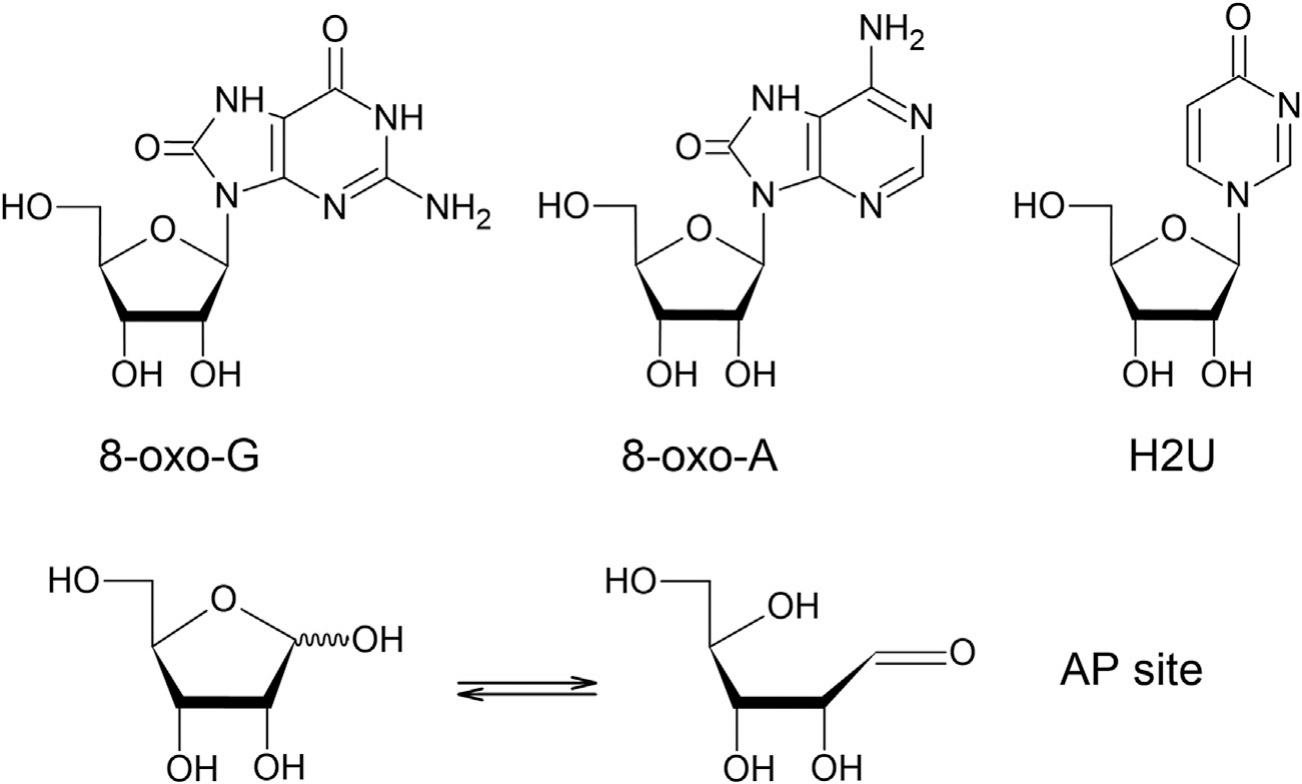
Chemical structures of the representative oxidative derivatives. 8-oxo-G (8-oxo-7,8-dihydro-guanosine), 8-oxo-A (8-oxo-7,8-dihydro-adenosine), H2U (4-pyrimidinone ribofuranoside), and AP site (apurinic/apyrimidinic site). AP site is tautomerized to give rise to two forms.

To our knowledge, the first investigation to quantify 8-oxo-G as a RNA oxidative derivative was reported in 1989 (Fiala et al., 1989). 8-oxo-G in rat liver RNA was elevated by treatment with a hepatocarcinogen, 2-nitropropane. It has been generally thought that 8-oxo-G is the most abundant oxidative derivative in RNA oxidation. In addition, there have been several assays developed to quantify 8-oxo-G: 1) high-performance liquid chromatography coupled with an electrochemical potential detector (Floyd et al., 1986; Shigenaga et al., 1989), 2) liquid chromatography/mass spectrometry or gas chromatography/mass spectrometry (Dizdaroglu et al., 2001; Weidner et al., 2011; Weimann et al., 2002), and 3) immunological assays using an anti-8-oxo-G antibody, including enzyme immunoassay, northern blotting, and immunoprecipitation (*e.g.* (Görg et al., 2008; Shan and Lin, 2006)). Therefore, most studies have assessed RNA oxidation based on 8-oxo-G production, while there have been no detailed investigations about the oxidation of G to 8-oxo-G. Conversely, DNA oxidation studies suggest that the hydroxyl radical does not react with the guanine base by electron transfer but by direct addition of the radical onto the double bonds of guanine at the C8 and C4 positions. Subsequently, the former guanine adduct that is attacked at the C8 position reacts with oxygen to give rise to 8-oxo-G (Figure 2) (Candeias and Steenken, 2000). *In vivo* study on iron metabolism revealed that 8-oxo-G in human urine was correlated with the expression levels of ferritin, transferrin, and transferrin saturation in the plasma (Cejvanovic et al., 2018). Several reports showed a positive correlation between RNA oxidation and cellular iron levels (Honda et al., 2005; Zinskie et al., 2018). Using immunoprecipitation with anti-8-oxo-G antibodies, oxidized mRNA was isolated from AD brains. Microarray analyses of the oxidized mRNA showed that the oxidation levels were dependent on the mRNA species; for instance, Cu/Zn superoxide dismutase 1 or presenilin 1 was found to be highly oxidized compared to other genes (Shan et al., 2003; Shan and Lin, 2006). Subsequent studies consistently showed selective mRNA oxidation in ALS patients’ brains, although which mRNAs were highly oxidized seemed to be different between ALS and AD (Shan et al., 2003; Chang et al., 2008). Selective oxidation of mRNA was also reported in ripened seed (Bazin et al., 2011), and other pathologies including ammonia toxicity (Görg et al., 2008) and formaldehyde exposure (Gonzalez-Rivera et al., 2020). Of note, highly oxidized mRNA species in their studies seemed to be inconsistent, suggesting that further study is necessary to clarify determinants to regulate selective mRNA oxidation.

### Abasic Site

Abasic site is a sugar moiety formed after a nucleobase is cleaved off. It is estimated that the abasic sites in genomic DNA are the most abundantly generated oxidative modification. It yielded approximately 10,000 to 50,000 sites mediated by site-specific spontaneous reaction (Fresco and Amosova, 2017), enzymatic reaction (Dizdaroglu et al., 2017), and oxidative reaction (Greenberg, 2016) in a given day (Lindahl, 1993). In addition to the typical abasic site, which is termed the AP site, several different structures of abasic sites are generated by DNA oxidation, for instance 2-deoxypentos-4-ulose and 2-deoxyribonolactone (Greenberg, 2016). While these atypical abasic sites have not been identified individually in oxidized RNA, our previous study using an aldehyde-reactive probe (ARP) revealed that AP site is generated by different types of oxidative reactions, including the iron mediated Fenton reaction, γ-irradiation, and peroxynitrite *in vitro*, and in cell cultures after oxidative stress with H_2_O_2_ (Tanaka et al., 2011). As shown in Figure 2, there are two tautomers, one that closed ring form and another that gives an open ring form which gives rise to an aldehyde group. These results indicate that an AP site is a biomarker for RNA oxidation. Conversely, Leumann’s group have established a method for synthesizing RNA with an AP site at a specific location (Küpfer and Leumann, 2007a) and characterized their chemical property. Their studies revealed that the AP site in RNA was chemically more stable relative to that in DNA (Küpfer and Leumann, 2007b) and preferentially base-paired with dA or dC during reverse transcription (Küpfer et al., 2007). In order to measure the oxidation levels of individual RNA species including mRNA, our group isolated and quantified abasic RNA from total RNA by pull-down assay using ARP (Tanaka et al., 2011). While the mitochondrial mRNAs which are encoded in the mitochondrial genome had comparable oxidation levels to those of cytoplasmic mRNA under normal conditions, the mitochondrial mRNAs were oxidized distinctly higher than cytoplasmic mRNAs after H_2_O_2_ treatment. Consistent with the findings of selective mRNA oxidation monitored with 8-oxo-G (Shan and Lin, 2006; Görg et al., 2008; Bazin et al., 2011; Gonzalez-Rivera et al., 2020), the oxidation levels of mRNA based on the AP site quantity vary depending on mRNA studied. For instance, AP site levels of *Oma1* mRNA were found to be about five-fold higher than that of ribosomal protein *Rpl5* mRNA (unpublished data).

### Other Oxidative Derivatives

The 8-oxo-A is known to be an intermediate of adenosine oxidation by xanthine oxidase (Wyngaarden and Dunn, 1957), however, it was also identified by mass spectrometry in polyA after oxidative reactions (Figure 2) (Alexander et al., 1987). 8-oxo-A has been assumed to be a minor oxidized ribonucleoside relative to 8-oxo-G, since 8-oxo-dA was reported to be generated approximately one-tenth of 8-oxo-dG in DNA (Frelon et al., 2000; Choi et al., 2017). However, it was reported that 8-oxo-A adduct was increased in the most affected brain regions of late stage AD compared to the age-matched control subjects while 8-oxo-G adduct was decreased (Weidner et al., 2011). *In vitro* oxidoreduction of yeast RNA in the presence of NADH and K_3_Fe(CN)_6_ gave rise to the hydroxylated pyrimidines 5-OH-C and 5-OH-U in addition to 8-oxo-G and 8-oxo-A (Yanagawa et al., 1990; Yanagawa et al., 1992). Due to having lower redox potentials, those oxidized ribonucleosides would be susceptible to further oxidative modifications; for example, 5-guanidinohydantoin was identified after a metal ion catalyzed oxidation of 8-oxo-G (White et al., 2005). In a separate study, Crean et al. reported the generation of spiroiminodihydantoin from 8-oxo-G by a carbonate radical anion, using 2′,3′,5′-tri-O-acetyl-8-oxo-G instead of 8-oxo-G (Crean et al., 2005). Oxidation mediated by manganese porphyrin generated spiroiminodihydantoin and 5-dehydroguanidinohydantoin as well (Tomaszewska-Antczak et al., 2015). Likewise, it was reported that oxidation of 8-oxo-A generated the quinoidal intermediate that tends to form adducts with a variety of nucleophilic compounds (Nilov et al., 2013).

Transfer RNA contains a variety of modified nucleotides, some of which serve as targets for oxidative modifications. The oxidative desulfration of thiouridine which contains sulfur in place of carbonyl oxygen atom has been investigated in detail. Nawrot’s group reported that the 2-thiouridine (S2U) is oxidized *via* a sequence of intermediates starting with sulefenic acid (-SOH), and then becoming sulfinic acid (-SO_2_H), and then sulfonic acid (-SO_3_H), which is desulfrated to generate 4-pyrimidinone ribofuranoside (H2U) (Figure 2) and uridine by H_2_O_2_ treatment (Sochacka et al., 2011) under physiological pH conditions (Sochacka et al., 2013) and cytochrome *c* (cyt *c*) mediated peroxidation (Sierant et al., 2018). Conversely, selenouridine which contains selenium instead of sulfur is oxidized in a similar pathway; however Payne et al. investigated redox chemistry between 2-selenouridine and 2-thiouridine to find that oxidized selenouridine was more prone to be reduced in the presence of antioxidants such as glutathione and ascorbate than oxidized thiouracil since selenouridine was highly resistant to oxidative deprivation of selenium which is the key atom to catalyze redox reaction (Payne et al., 2017).

### Cross-Linking

There have been numerous studies regarding RNA-protein artificial cross-linking, mainly for mapping between RNA and its associated RNA binding proteins by means of site-specific probe insertion and UV irradiation (*i.e.* (Van Nostrand et al., 2016; Lin and Miles, 2019; Sharma et al., 2021)). For pathophysiological cross-linking, Mirzaei and Regnier reported RNA and protein cross-linking in yeast after H_2_O_2_ treatment (Mirzaei and Regnier, 2006). Proteomic mass spectrometric analysis for the isolated carbonylated proteins showed that the cross-linking was mainly between rRNA and ribosomal proteins at multiple sites. Cross-links were formed mainly between lysine, arginine, methionine, and tyrosine residues in ribosomal proteins, and guanine residue in rRNA. To this end, we have shown the *in vitro* oxidative cross-linking between tRNA and cyt *c* (Tanaka et al., 2012). Cyt *c* catalyzes the peroxidation of mitochondrial membrane lipids in the presence of H_2_O_2_ (Bayir et al., 2006; Yin et al., 2017) and exhibiting binding affinity between RNA and the heme *c* moiety. In this case, G appeared preferentially subject to oxidative modifications, including depurination and cross-linking, while the cross-linked structure between ribonucleotide and amino acid has not been identified (Tanaka et al., 2012). Thus, although there have been few studies in this field until now, it would be possible that oxidative stress induces RNA-protein cross-linking, which may contribute to impaired translation, deregulation of RNA binding proteins, or deteriorated protein homeostasis due to protein aggregation.

### Strand Scission

Strand scission would be one of the major outcomes observed due to RNA oxidative modification. Approximately 40% of *in vitro* reactions between hydroxyl radicals and RNA strands lead to strand scission, in which nucleobase radicals are primarily generated, followed by H-abstraction from ribose rings, and resulting in a strand break (Lemaire et al., 1984; Jones and O'Neill, 1990). Joyner et al. investigated the mechanism of oxidative RNA cleavage and formation of 3′- and 5′-overhangs using MALDI-TOF MS (Joyner et al., 2013). HIV-1 Rev Response Element was fragmented at various sizes by oxidation in the presence of ascorbate, H_2_O_2_, and Fe(II)-EDTA. The cleavage site and chemical structure of the terminal overhangs were identified by their molecular weights. In addition to 3′-PO_4_, 2′,3′-cyclic PO_4_ and 3′-phoshoglycolate were the main 3′-end structures, while 5′-PO_4_ and 5′-OH were the main 5′-end structures. Based on previous DNA studies, it was deduced that there are three different modes of cleavage reactions, hydrolysis, H-abstraction, and 2′-OH mediated transesterification: 3′-PO_4_ was generated by the hydrolytic process; 3′-phosphoglycolate formation occurred by oxidative 4′-H abstraction in the ribose moiety, and base 2-propenal was generated as a byproduct. In addition, 2′,3′-cyclicPO_4_ could be generated due to 2′-OH mediated transesterification (Joyner et al., 2013). Ingle et al. investigated oxidative cleavage in the GUA base triple in rat sarsin/ricin loop RNA. The reactivity of the GUA triplet with sodium borohydride, and the products analyses showed that the aldehyde moiety was generated at the 5′-end possibly due to 5′-hydrogen abstraction (Ingle et al., 2014). To date, several nucleases are known to cleave DNA 3′-phosphoglycorate (Kuo et al., 1993; Inamdar et al., 2002; Povirk et al., 2007), however no ribonuclease to cleave RNA 3′-phosphoglycorate has been reported. It may be important to investigate this enzyme activity for understanding RNA homeostasis during oxidative stress.

## Effects of RNA Oxidation on Translation

To our knowledge, since inhibition of the aminoacylation activity by RNA oxidation was first reported in 1959 (Preiss et al., 1959), translational activity has been investigated to assess biological consequences of RNA oxidation. Here we review translational alternation by oxidation of mRNA and rRNA mainly modified with 8-oxo-G or abasic site. As for tRNA, we review here the effects of oxidative desulfration of thiouridine.

### tRNA Oxidation

*In vitro* aminoacylation of tRNA after oxidation were substantially reduced for glutamic acid, glutamine and lysine compared to other amino acids. Their tRNAs contain thiouridine residues, which were desulfurated (Rao and Cherayil, 1974). It was also reported that desulfrated tRNAs substantially reduced ribosome binding depending on the mRNA template (Walker and RajBhandary, 1972). Since thiolated uridines are frequently located at the wobble position, the first nucleotide of anticodon, it is of interest how codon-anticodon interaction is changed by thiouridine desulfration. To this end, Sochacka et al. investigated base pairing with S2U and H2U which were incorporated into RNA duplex oligonucleotides with A or G as the complementary nucleotides. Based on thermodynamic analyses and the computational calculation, it was found that the H2U-G paired duplex was thermodynamically more stable than the H2U-A paired duplex, possibly because there are two putative hydrogen bonds between H2U and G, whereas there is only one hydrogen bond between H2U and A. Thus, although S2U is known to form a base pair with A, H2U seems to base pair with G, which may alter decoding kinetics by oxidation of thiouridine at the wobble position (Sochacka et al., 2015).

### mRNA Oxidation

Previously we reported on translational inhibition of oxidized mRNA *in vitro* (Tanaka et al., 2007). Using cell free translation systems, oxidized luciferase mRNA was found to compromise translational activity, even if the mRNA was incubated with only iron under aerobic conditions, and in the absence of ascorbate and H_2_O_2_. In addition, we observed the accumulation of short polypeptides, possibly due to premature termination as they were mostly N-terminal fragments. These fragments were also generated in cell cultures transfected with the oxidized mRNA or treated with paraquat following transfection with non-oxidized mRNA. But the peptide fragments were observed only in the presence of a proteasome inhibitor, MG132. Together, these observations indicate that the abnormal polypeptides were readily eliminated by proteasomes. In addition, we found that the catalytic activity of the full-length luciferase generated using oxidized mRNA is compromised, suggesting that the protein structure so generated was altered (Tanaka et al., 2007). Consistent with the notion that protein synthesis using oxidized mRNA leads to the generation of abnormal proteins, overexpression using oxidized mRNA encoding EGFP in HEK293 cells has been shown to generate an EGFP protein that aggregated in abnormal cytoplasmic foci. This observation indicates that EGFP generated with oxidized mRNA tended to be aggregation-prone, possibly due to structural changes caused by miscoding (Shan et al., 2003). Together, these studies demonstrated that oxidized mRNA expression in cells would lead to abnormal polypeptide production, although it is not known exactly how it occurred.

Recent studies have expanded mechanistic understanding of the above observations using mRNA templates which incorporated 8-oxo-G, AP site, or other oxidative nucleotides at a specific position (Simms et al., 2014; Calabretta et al., 2015; Hoernes et al., 2018a; Hoernes et al., 2018b; Thomas et al., 2019). Zaher’s group revealed, when an mRNA oligomer was incubated with wheat germ or an *E. coli* derived *in vitro* translation system, the translation process would stop or take an extremely long pause at the 8-oxo-G site wherever it was located within a triplet codon (Simms et al., 2014). This *in vitro* study demonstrated that 8-oxo-G in mRNA largely inhibits translation elongation. Later it was found that inhibition of translation elongation is a common dysfunctional mechanism for other oxidized derivatives. Calabretta et al. investigated amino acid incorporation at the lesioned site, where 8-oxo-G, 8-oxo-A, 5-OH-C, or 5-OH-U was incorporated into a synthetic mRNA oligomer using rabbit reticulocyte lysate. They found the translated products were substantially truncated at the lesioned site where 8-oxo-G, 8-oxo-A, 5-OH-C, or 5-OH-U was inserted, while 1,N6-ethenoAdo, 3,N4-ethenoC, or abasic site showed no translation elongation at the insertion site (Calabretta et al., 2015). Thus, all the oxidized ribonucleosides tested appear to cause translation arrest. In this situation, the No-Go decay system is activated and endoribonucleolytic degradation of the mRNA stalled on the ribosome triggers downstream quality control pathways (Simms et al., 2017). Yeast mutants dom34Δ and xrn1Δ, which correspond to the ribonucleolytic activity, showed an increase of the 8-oxo-G levels in polyA RNA. This observation indicates that once ribosomes are stalled at the oxidative modified sites of mRNA, a No-Go decay enzyme complex is recruited to the sites and starts to degrade of the mRNA (Simms et al., 2017; Yan et al., 2019) interacting with Rps3/uS3 at the entry tunnel of the ribosomal small subunit (Simms et al., 2018).

In terms of miscoding, base-pairing against 8-oxo-G on the ribosome has been investigated using an *in vitro* system (Thomas et al., 2019). 8-oxo-G at the first codon preferentially base paired with C while 8-oxo-G at the second codon tended to base pair with A taking presumably *syn* conformation. Under translation error prone conditions, such as treatments with paromomycin, streptomycin or having an error-prone ribosome mutant, increased the rate of peptide bond formation in both base-pairings. Thus, these findings provide evidence that 8-oxo-G in mRNA has potential to cause amino acid misincorporation during the coding process, despite the fact that the peptide bond formation rate was very low (Thomas et al., 2019). Erlacher’s group investigated translation products from mRNA containing non-standard codon nucleotides including AP site using mass spectrometry to identify decoding amino acid (Hoernes et al., 2018a). When one AP site was inserted in the third codon (A-U-AP site) in a synthesized reporter mRNA, the *in vitro* bacterial translation system translated the codon into predominantly as Ile (95%), but also a small amount of Met (5%). Even though the translation was not efficient, the AP site was found to be decoded in certain nucleotides but not a single nucleotide. Conversely, HEK293T eukaryotic cells did not translate the modified codon (Hoernes et al., 2018a). In addition, they investigated the translational termination at stop codons with several modified ribonucleotides including AP site as well (Hoernes et al., 2018b). Triple AP sites completely inhibited the peptide release in both bacterial and eukaryotic release factors. Insertion of an abasic site at the second or third codon inhibited peptide release; the exact influence was significantly different based on the release factor and the abasic position (Hoernes et al., 2018b).

### rRNA Oxidation

Honda et al. investigated translational activity of *in vitro* oxidized ribosomes. As expected, iron catalyzed oxidation significantly reduced the translational activity, which was measured by radiolabeling amino acid incorporated in the proteinaceous fraction (Honda et al., 2005). The peptidyl transferase center (PTC) region in the ribosome plays a pivotal role in aminoacyltransferase activity, where it is mediated by highly structured rRNA but not associated ribosomal proteins. Polacek’s group chemically synthesized in *E. coli* large rRNA in which certain ribonucleotide was replaced with an abasic site in the PTC region and integrated to reconstitute a whole ribosome. It was found that translational activity, including peptide bond formation, was reduced by substitution at A2450, A2451, or C2063, whereas substitution in positions, U2585 and U2506, increased translational activity (Erlacher et al., 2005; Erlacher et al., 2006; Amort et al., 2007; Chirkova et al., 2010). Schrode et al. investigated translational activity of 16S rRNA modified by replacing the AP sites at A1492 and A1493, which are critical for A-site binding of aminoacyl tRNA, and this was found to abolish translational activity (Schrode et al., 2017). Willi et al. extended the investigation using hydroxylated ribonucleotides (Willi et al., 2018). Large 23S rRNA which was individually substituted to 8-oxo-G (G2447), 8-oxo-A (A2451 or A2602), 5-OH-C (C2063) or 5-OH-U (U2585 or U2506) in the PTC region was synthesized and reconstituted the ribosome. It turned out that 8-oxo-A2451 caused slow peptide bond formation and 5-OH-U2585 interfered with A-site tRNA accommodation, while 5-OH-U2506, 8-oxo-A2602, and 8-oxo-G2447 did not affect the translational activity. Conversely, 5-OH-C2063 facilitated translation (Willi et al., 2018). Thus, these results suggested that depurinated or oxidative modifications in rRNA do not necessarily impair ribosomal translational activity, but rather the effect is dependent on the modified position.

## Oxidatively Modified RNAs as Potential Signaling Modulators

### Oxidized Ribonucleoside as Redox Catalyst and Antioxidant

Yanagawa et al. reported that hydroxylated ribonucleosides, 5-OH-C, 5-OH-U, and 8-oxo-G, are capable of facilitating the oxidoreduction between K_3_Fe(CN)_6_ and NADH. The redox potentials (E7) for 5-OH-C, 5-OH-U, 8-oxo-A and 8-oxo-G were found to be 0.42, 0.44, 0.72, and 0.38 V, respectively. Considering the redox potentials of K_3_Fe(CN)_6_ (0.42 V) and NADH (−0.35 V), it is reasonable that 5-OH-C, 5-OH-U, and 8-oxo-G but not 8-oxo-A could catalyze the oxidoreduction between the two compounds (Yanagawa et al., 1992). It is possible that those oxidized ribonucleosides are more reactive in the tested conditions than the standard ribonucleosides since the redox potentials (E7) of A and G were 1.42 and 1.29 V, respectively (Steenkcn and Jovanovic, 1997).

As mentioned above, due to the low redox potential, 8-oxo-G is readily oxidized by the reaction with ROS (Crean et al., 2005; White et al., 2005), suggesting that this oxidized ribonucleoside could function as an ROS absorber. Lee et al. reported that levels of hydroxyl radical generated by Fenton reaction, peroxynitrite, and superoxide anion could be reduced by co-incubation with 8-oxo-G or 8-oxo-dG, equivalent or even more efficient than *N*-acetyl cysteine (Lee et al., 2013). Thus, due to its relatively low redox potential, oxidized ribonucleoside such as 8-oxo-G could function as a catalyst for the oxidoreduction within a certain range of redox potentials or as an antioxidant to remove the ROS in cells as well as in biofluid.

### Free 8-Oxo-GTP as a Signaling Modulator

Although 8-oxo-GTP is hydrolyzed to 8-oxo-GMP by MutT/MTH1 (Taddei et al., 1997), the cytoplasmic 8-oxo-GTP pool increased during oxidative stress (*i.e.* approx. 4 and 9 pmol/mg protein in control and stress conditions in PC12 cells, respectively (Bolin and Cardozo-Pelaez, 2009)). Chung’s group investigated the effects of free 8-oxo-GTP on several small G-proteins: in HEK293 cell lysate the recombinant Ras was activated and enhanced the downstream Ras-ERK signaling pathway by 8-oxo-GTPγS more efficiently than GTPγS, the unhydrolyzable analog of GTP. In contrast, recombinant Rac1 and Cdc42 were activated by GTPγS, while they were inactivated by 8-oxo-GTPγS (Yoon et al., 2005). Additionally, the production of NADPH oxidase-derived superoxide anion was elevated by GTPγS, while it was decreased by 8-oxo-GTPγS in human neutrophil lysate activated by phorbol myristate acetate (PMA). Consistently, activity of Rac1 which is bound to the active form of NADH oxidase was increased by GTPγS but decreased by 8-oxo-GTPγS, suggesting that 8-oxo-GTP is a negative modulator to inflammatory responses involving the NADPH oxidase and Rac1 complex (Kim et al., 2007).

In the presence of nitric oxide, the soluble guanylyl cyclase (sGC) is activated to catalyze GTP to cyclic-GMP (cGMP), the second messenger for various downstream signaling for proliferation, synaptic plasticity, vasodilation, and platelet aggregation. Bolin and Cardozo-Pelaez investigated the effect of 8-oxo-GTP on sGC (Bolin and Cardozo-Pelaez, 2009). They observed that when purified sGC was incubated in the presence of a NO donor with different doses of 8-oxo-GTP, cGMP production was decreased in a manner of reducing Vmax without altering the value of Km. Conversely, sGC activity was decreased in cultured cells after a copper/ascorbate mediated Fenton reaction, a condition which did not induce the reduction of the intracellular GTP pool or oxidative damage of the protein. Instead, it would be, in part, due to the increase in intracellular 8-oxo-GTP (Bolin and Cardozo-Pelaez, 2009).

### Oxidized Mitochondrial RNA as an Inflammation Modulator

Oxidized mitochondrial DNA transfected into the cytoplasm or injected into animal tissue, induced an inflammatory response mediated by the NLRP3 inflammasome (Collins et al., 2004; Shimada et al., 2012). Saxena et al. investigated the immune response by oxidized mitochondrial RNA (mtRNA) in a mouse bone marrow derived macrophage (Saxena et al., 2017). When oxidized mtRNA isolated from H_2_O_2_ treated HA1 hamster fibroblast cells was transfected, production of proinflammatory cytokines, IL-6, MCP-1, and type I interferon were found lower than those of control mtRNA isolated from untreated cells. In contrast, when oxidized mtRNA derived from H_2_O_2_ treated THP-1, human monocyte cells were transfected to the differentiated THP-1 induced by PMA, the inflammatory response to induce IL-6 and TNF-α was more activated relative to that of control mtRNA (Ilic et al., 2019). Taken together, it seems to be inconclusive whether oxidized mtRNA in cytoplasm shows differential effects on inflammatory response compared to control mtRNA. However, it is possible that oxidation of mtRNA may acquire differential inflammatory responses under certain conditions.

### Oxidized RNA in Apoptotic Pathways

Wang et al. investigated genome-wide analysis to identify selectively oxidized miRNAs under oxidative stress conditions in a rat heart cell line. They revealed that miR-184 was one of the highly oxidized miRNAs. The oxidized miR-184, but not non-oxidized miR-184, preferentially interacted with two anti-apoptotic genes, Bcl-xl and Bcl-w mRNA at 3′-UTR and lead to their downregulation (Wang et al., 2015). Although the oxidized positions in the miRNA have not been identified, the putative mismatch between oxidized miRNA184 and the target 3′-UTR indicated that several G residues in miRNA184 were base-paired with A, the nucleoside that base paired with 8-oxo-G (Koga et al., 2013). It should be pointed out that those putative oxidized G sites are mostly located in the G repeat sequence, which is more susceptible to oxidation compared to a single G due to lower redox potential (Burrows and Muller, 1998; Margolin et al., 2008). Degradation of anti-apoptotic genes due to binding the oxidized miRNA would induce an acceleration of cellular apoptosis. This observation suggests a molecular mechanism by which oxidative stress induces cellular apoptosis. Moreover, this would be the case *in vivo* because injection of oxidized miR-184 into the heart of an ischemia/reperfusion animal model potentiated the reduction of Bcl-xl and Bcl-w, while increasing the apoptotic cells and infarct size compared to control miR-184 injection (Wang et al., 2015). Thus, miRNA184 acquires the novel function of binding to new target mRNAs by oxidative modification which in turn regulates apoptotic signaling.

Sekiguchi’s group has identified several proteins that can bind to oxidized RNA from bacteria and mammalian cells by screening RNA oligonucleotides containing 8-oxo-G as a probe; polynucleotide phosphorylase 1 (PNPase) (Hayakawa et al., 2001), Y box-binding protein (YB-1) (Hayakawa et al., 2002), heterogeneous nuclear ribonucleoprotein splice form D0 (HNRNPD or AUF1) and C1 (HNRPNC) (Hayakawa et al., 2010; Ishii et al., 2015), and poly(C) binding protein (PCBP) 1 (Ishii et al., 2018) and PCBP2 (Ishii et al., 2020). *In vitro* binding tests showed that PCBP1 and PCBP2 bind to a highly enriched 8-oxo-G oligo RNA in comparison to AUF1, which efficiently binds to a single 8-oxo-G containing oligo RNA. PCBP1 deficient HeLa cells showed lower capase-3 activation and poly (ADP-ribose) polymerase 1 (PARP1) cleavage after H_2_O_2_ treatment leading to a higher survival rate. In addition, PCBP1 mutant in KH1 domain showed deficiency both in 8-oxo-G binding and PARP-1 cleavage (Ishii et al., 2018). In contrast, PCBP2 deficient HeLa cells exhibited growth retardation, with increasing caspase-3 activity, cleaved PARP, and apoptosis under oxidative stress (Ishii et al., 2020). Together, these results indicate that PCBP1 and PCBP2 modulates apoptotic signaling in an opposite direction after binding to highly oxidized RNA. While there are many factors known to be involved in regulating cellular apoptosis, the PCBP1 and PCBP2 may involve in mediating the apoptosis in response to cellular RNA oxidation.

It is well established that cyt *c* catalyzes peroxidation through the heme iron. To this end, we have reported that cyt *c* readily oxidized tRNA to form a cross-link in the presence of H_2_O_2_ (Tanaka et al., 2012), partly due to cyt *c* binding to tRNA *via* heme *c* domain (Gorla and Sepuri, 2014). Under these conditions, the cyt *c*-tRNA complex would be released from a lipid vesicle composed of cardiolipin, the main mitochondrial membrane component. As a result, the oxidative cross-linking of cyt *c* with tRNA could facilitate the release of cyt *c* from mitochondria, and activate cellular apoptosis (Tanaka et al., 2012). Nevertheless, it should be pointed out that Mei et al. had reported that the cyt *c*-tRNA non-covalent complex could bind to Apaf1 (Mei et al., 2010) and inhibit caspase activation.

### miRNA Processing Regulated by Its Oxidation

Apurinic/apyrimidinic endonuclease1 (APE1) is long known as the endodeoxyribonuclease which cleaves off DNA strand specifically at the 5′ side to the AP site during the process of DNA base excision repair. Later, this enzyme was found to be able to cleave c-myc mRNA (Barnes et al., 2009) and was confirmed to be an endoribonuclease that cleaves at the RNA abasic site (Berquist et al., 2008). Moreover, Tell’s group reported that APE1-knockdown cells contain an increased level of oxidized RNA, indicating that APE1 plays a critical role in clearance of oxidized RNAs (Vascotto et al., 2009). Recently, they identified the interacting miRNAs which are corelated to the cancer development and characterized their maturation processes (Antoniali et al., 2017). Among them, miR-221 and miR-222 which are involved in the expression of PTEN, the tumor suppressor gene, was upregulated in cells treated with H_2_O_2_. Their further analysis revealed that the precursors, pri-miR221 and pri-miR222 were elevated while their mature forms were decreased, either by APE1 knockdown, APE1 mutant overexpression, or the endoribonuclease inhibitor. Together, their findings suggest that APE1 regulates the miRNA processing, possibly through AP endoribonuclease activity. As a matter of fact, APE1 knockdown cells increased the oxidative AP site levels of miR221, while the revertant cells due to APE1 overexpression returned to control levels. Certainly, APE1 knockdown cells upregulated PTEN expression, and the expression levels of APE1 were positively correlated with those of miR221 but reciprocally correlated with those of PTEN in human tumor tissue (Antoniali et al., 2017). These results indicated that the miRNA processing and maturation are regulated through AP site formation in the miRNAs and their degradation by APE1.

## Effects of Enzymatically Created RNA AP Site

### AP Sites for Antiviral Defense

AP site formation by depurination is not only due to RNA oxidative modification by ROS, but is also known to be created by proteins. Here we discuss mimic oxidative modification and its biological consequences. Endo and Tsurugi discovered that ricin A-chain depurinated 28S rRNA at a conserved A4324 in the sarcin/ricin loop (Endo et al., 1987; Endo and Tsurugi, 1987), which has an essential role on translocation step associated with elongation factor binding (Montanaro et al., 1975; Shi et al., 2012). To date, several proteins and families with the RNA N-glycosidase activity have been identified mainly in plants, fungi, and bacteria. These ribosome inactivating proteins (RIPs) caused complete inactivation of ribosomal function leading to cell death, although it is arguable whether the potent cytotoxicity of RIPs is only due to the *N*-glycosidase activity (Hudak et al., 2004). Pokeweed antiviral protein (PAP) was first characterized as an antiviral protein because plant virus infection was inhibited by co-inoculation (Wyatt and Shepherd, 1969; Tomlinson et al., 1974). PAP depurinates A residues not only in the host rRNA but also in several viral RNAs following recognizing the cap structure of the RNAs (Hudak et al., 2000; Hudak et al., 2002). As mentioned in the previous section, depurinated viral RNA such as Bromo mosaic virus RNA3 was not translated properly and rapidly degraded by No-Go decay system (Gandhi et al., 2008). Although the molecular mechanism to recognize viral RNA has not been fully understood yet, depurination of both rRNA and viral mRNA in the infected cells seems a quite potent modification to cause malfunction on viral protein synthesis. Organisms may have evolved to cope with viral infection by taking advantage of the same chemical modification as occurred in oxidative stress to the pathogen RNA and eliminating it using the No-Go decay RNA quality control system.

### AP Sites to Regulate R-Loop

R-loop is a three-stranded nucleic acid structure. It consists of a DNA-RNA hybrid and displaced single-stranded of DNA. It is formed during transcription when the nascent RNA reanneals with the template DNA strand. R-loops play vital roles in regulating gene expression, DNA replication, and DNA and histone modifications. It is thought to regulate several cellular processes including immunoglobulin class switch recombination, transcriptional gene expression, mitochondrial DNA replication, and epigenetic modification. Conversely, dysregulated R-loop formation causes DNA damage and genome instability, and lead to a number of human diseases, including neurological disorders, cancer, and autoimmune diseases (see review (García-Muse et al., 2019; Hegazy et al., 2020)). However, it is not well understood how R-loop structure is stabilized or resolved. Recently proteomic analysis for the binding factors on R-loop revealed hundreds of proteins involved in RNA binding, splicing, helicase, transcription termination, and telomere regulation (Wang et al., 2018). Among them, the DNA damage excision repair enzymes, APE1, and methylpurine DNA glycosylase (MPG) were further investigated (Liu et al., 2020). It was found that RNA substrates of APE1 and MPG co-localized to the R-loop regions based on genome-wide analysis. The MPG knockdown cells temporally decreased abasic RNA as well as R-loop regions. Purified MPG had a *N*-glycosylase activity on the RNA strand in the RNA-DNA duplex *in vitro*. In addition, APE1 incises the abasic RNA strand in the RNA-DNA duplex (Liu et al., 2020). These findings suggested that the RNA strand in the R-loop is depurinated by MPG and cleaved off at the AP site by APE1. It is possible that these two enzymes collaboratively regulate the R-loop stability via the abasic site formation and the ribonucleolytic cleavage of the abasic RNA strand.

The scheme in Figure 1 depicts the summary of potential pathological and physiological effects of oxidized nucleoside, and/or RNA. While these oxidative modifications not only could lead to impairing their normal physiological functions, they could also modulate cellular signaling pathways, such as inducing cellular apoptosis in response to oxidative stress.

## Perspectives

The level of oxidized cellular RNA is higher than that of genomic DNA under oxidative stress conditions, *in vitro* and *in vivo*, observed with H_2_O_2_ treatment (Hofer et al., 2005), UV irradiation (Wamer and Wei, 1997), or treatment with hepatocarcinogen 2-nitropropane (Fiala et al., 1989). These effects, in part, are due to the DNA protection exerted by nucleosomal histone proteins against iron-mediated damage (Enright et al., 1992) and the presence of robust DNA oxidation damage repair/degradation systems (Chapman et al., 2012; Chatterjee and Walker, 2017). On the other hand, substantial evidence shows the presence of enzyme systems that eliminate RNA oxidation; APE1 is an endonuclease that breaks down the abasic RNA (Vascotto et al., 2009). PNPase has been identified as a 8-oxo-G binding protein (Hayakawa et al., 2001), and later it was suggested to be involved in degradation of oxidized RNA (Wu and Li, 2008). MutT/MTH1 is a pyrophophatase that catalyzes the hydrolysis of 8-oxo-GTP to 8-oxo-GMP to exclude incorporation of the oxidized ribonucleotide into RNA (Taddei et al., 1997). Moreover, the No-Go decay system, which degrades many different types of abnormal mRNA, once they are stalled on the ribosome, plays a pivotal role on the elimination of oxidized mRNA (Gandhi et al., 2008; Yan and Zaher, 2019). Therefore, it is unlikely that high RNA oxidation levels are due to the absence of oxidized RNA removing systems. Instead, it may be possible that biosystems have evolved to be tolerant of RNA oxidation relative to DNA oxidation. Oxidized RNAs may not be eliminated instantly in cells. Recent studies have revealed emerging roles for oxidized RNA as signaling modulators in response to cellular oxidative stress. The level of oxidized RNAs might be elaborately controlled to prevent a global protein homeostasis crisis, as well as to accommodate the regulatory functions of oxidized RNA. Thus, it is clear that further research on oxidized RNA is essential.

## Abbreviations

AD, Alzheimer’s disease; A, adenosine; ALS, amyotrophic lateral sclerosis; APE1, apurinic/apyrimidinic endonuclease1; AP site, apurinic/apyrimidinic site; ARP, aldehyde reactive probe; Cyt c, cytochrome c; C, cytidine; EGFP, enhanced green fluorescent protein; G, guanosine; H2U, 4-pyrimidinone ribofuranoside; MPG, methylpurine DNA glycosylase; mtRNA, mitochondrial RNA; NLRP3, (NOD)-like receptor protein 3; RIP, ribosome inactivating protein; PAP, pokeweed antiviral protein; PARP-1, poly(ADP-ribose) polymerase 1; PCBP, poly(C) binding protein; PD, Parkinson’s disease; PMA, phorbol myristate acetate; PNPase, polynucleotide phosphorylase1; PTC, Peptidyl Transferase Center; sGC, soluble guanylyl cyclase; ROS, reactive oxygen species; S2U, 2-thiouridine; U, uridine; 8-oxo-G, 8-oxo-7,8-dihydro-guanosine; 8-oxo-dG, 8-oxo-7,8-dihydro-deoxyguanosine; 8-oxo-A, 8-oxo-7,8-dihydro-adenosine.
